# Stepwise administration of de novo ketogenic parenteral nutrition for refractory epilepsy during prolonged bowel rest following duodenal perforation

**DOI:** 10.1002/jpr3.70293

**Published:** 2026-10-30

**Authors:** Zaid Sheikh, Sarika Sewak, Danielle N. Mein, Joyce H. Matsumoto, Alvin P. Chan

**Affiliations:** ^1^ Division of Pediatric Gastroenterology, Department of Pediatrics Holtz Children's Hospital, UM Miami Florida USA; ^2^ Department of Clinical Nutrition UCLA Mattel Children's Hospital, UCLA Los Angeles California USA; ^3^ Division of Pediatric Neurology, Department of Pediatrics UCLA Mattel Children's Hospital, UCLA Los Angeles California USA; ^4^ Division of Pediatric Gastroenterology, Hepatology and Nutrition, Department of Pediatrics UCLA Mattel Children's Hospital, UCLA Los Angeles California USA

**Keywords:** gastrojejunostomy tube, ketosis, lipids, seizure

## Abstract

Ketogenic diet is an established therapy for pediatric patients with intractable epilepsy. By shifting energy metabolism from glucose to fat, the diet increases the production of ketone bodies, which alters neuronal excitability and modulates neurotransmitter signaling, thereby raising the seizure threshold. In critically ill children who are unable to tolerate enteral feeds, ketogenic parenteral nutrition (kPN) offers a valuable alternative to the standard ketogenic diet. However, its use has been underutilized and challenged by a lack of standardized protocols. In this report, we present a stepwise approach for initiating kPN in a ketogenic diet‐naive toddler with refractory epilepsy and bowel dysfunction.

## 1 INTRODUCTION

The ketogenic diet is an effective treatment for drug‐resistant epilepsy and status epilepticus.^1^ Its mechanism of action involves a metabolic shift from glucose to fat as the primary energy source, leading to increased production of ketone bodies from acetyl coenzyme A.^2^ The three molecules functionally grouped as ketone bodies are acetoacetate, β‐hydroxybutyrate (β‐OHB), and acetone. Elevated ketone bodies increase the seizure threshold by altering neuronal excitability and modulating neurotransmitter signaling, favoring inhibition over excitation.^2^ β‐OHB is monitored because it is the predominant circulating ketone in nutritional ketosis and it has reliable point‐of‐care assays. In select cases, ketogenic parenteral nutrition (kPN) may be warranted for seizure control when enteral feeding is not tolerated, enteral access is lost, or bowel rest is necessary. Although the ketogenic diet is an established therapy for epilepsy, its use in the form of kPN remains underutilized due to limited clinical experience.^3^

## 2 CASE REPORT

A 14‐month‐old girl with infantile spasms underwent endoscopy for conversion to a standard gastrojejunostomy tube (Traditional G‐JET®, 14 Fr, 1.0 cm, 15 cm; Applied Medical Tech) for recurrent aspiration and gastric feeding intolerance. She had no immediate complications and was discharged home on full jejunal feeds. However, she presented to the emergency department 2 weeks later with status epilepticus and abdominal distention. She was found to have a duodenal perforation, likely from pressure necrosis by the feeding tube.

She underwent emergent exploratory laparotomy and perforation repair. The postoperative course was complicated by prolonged ileus and refractory seizures despite optimization of antiseizure therapy with brivaracetam, cannabidiol, clobazam, perampanel, and rufinamide. A joint decision was made among the multidisciplinary team to initiate kPN for simultaneous management of worsening seizure burden, nutrition, and gastrointestinal dysfunction. Parenteral nutrition (PN) was transitioned to kPN and advanced conservatively because of her tenuous condition (Table 1). Lipid (SMOFlipid®; Fresenius Kabi) was advanced every 3 days to the highest possible ketogenic ratio (grams of lipid to grams of carbohydrate plus protein). Carnitine was supplemented to enhance lipid utilization. Dextrose in the PN was incrementally weaned from 5% to 0.1% (4.3–0.1 g/kg) while maintaining target caloric goals. To minimize extra carbohydrate exposure, brivaracetam was continued intravenously, and liquid suspensions of antiseizure medications were transitioned to tablet formulations when available, which included clobazam, perampanel, and rufinamide.

**Table 1:** Macronutrient composition and energy intake during ketogenic parenteral nutrition.

Day	Lipid	Dextrose % solution	Glycerol^a^	Carbohydrate^b^	Amino acid	Ketogenic ratio	Energy
	(g/kg)	(g/kg)	(g/kg)	(g/kg)	(g/kg)		(kcal/kg)
Baseline	2.5	5.0 (4.3)	3.2 (0.3)	46 (4.6)	2.2	0.4:1	49^c^
Day 1	3.0	2.5 (2.1)	4.3 (0.4)	25.5 (2.5)	2.2	0.6:1	47^c^
Day 4	3.2	1.5 (1.3)	4.7 (0.5)	17.5 (1.8)	2.2	0.8:1	47^c^
Day 7	3.6	0.5 (0.5)	5.2 (0.5)	9.9 (1.0)	1.8	1.3:1	47^c^
Day 10	3.7	0.1 (0.1)	5.4 (0.5)	6.3 (0.6)	1.8	1.5:1	46^c^

^a^ SMOF contains 2.5 g of glycerol per 100 mL.

^b^ Carbohydrate includes dextrose and glycerol.

^c^ Calories reflect 100% of resting energy expenditure measured by indirect calorimetry, with no stress or activity factors applied.

Ketosis, indicated by serum β‐OHB >1.0 mmol/L, was achieved by day 16. Serum glucose remained largely normal (90–101 mg/dL), except for four transient asymptomatic dips during initial dextrose weaning that resolved spontaneously (Figure 1). Aspartate aminotransferase (AST, 10–15 U/L) and triglyceride levels (92–116 mg/dL) stayed within range (Figure 1). A reduction (<50%) in seizure frequency was achieved. In total, kPN was administered for 38 days. As bowel function improved over time, kPN was transitioned to an enteral ketogenic diet via nasojejunal tube, with gradual advancement to a 3:1 ketogenic ratio. Seizure control was maintained during the transition and continued to improve on the enteral ketogenic diet, allowing gradual weaning of antiseizure medications.

**Figure 1:**
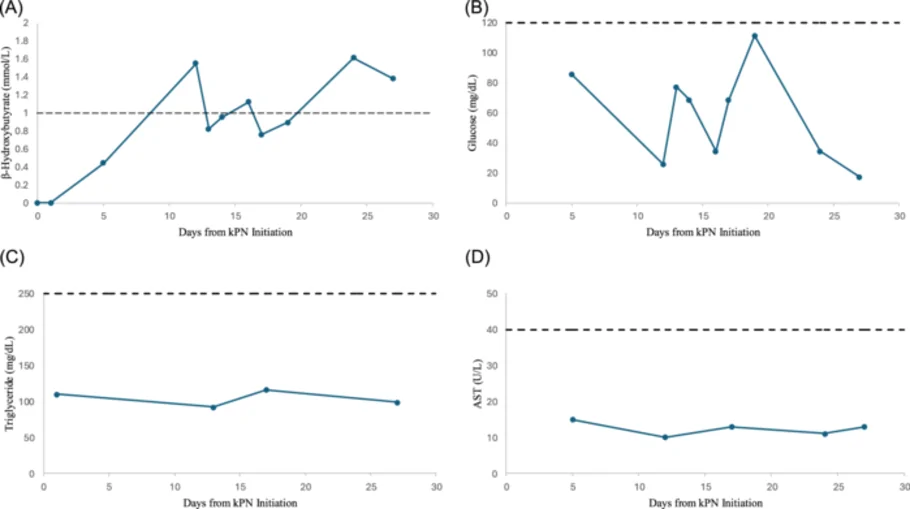
Laboratory monitoring for patient during ketogenic parenteral therapy. (A) β‐Hydroxybutyrate (mmol/L). (B) Glucose (mg/dL). (C) Triglyceride (mg/dL). (D) AST (U/L). AST, aspartate aminotransferase; kPN, ketogenic parenteral nutrition.

## 3 DISCUSSION

This report offers practical guidance for initiating kPN in ketogenic diet‐naïve pediatric patients unable to receive enteral nutrition, a scenario underrepresented in the literature. In our center, a serum β‐OHB > 1.0 mmol/L is used as a practical target to indicate stable ketosis during ketogenic therapy. Although serum β‐OHB concentrations >0.3 mmol/L exceed the normal range in individuals not following a ketogenic diet, levels just above this cutoff often slip in and out of ketosis. For this reason, our team uses β‐OHB > 1.0 mmol/L as the minimum threshold to maintain ketosis. Ketogenic diet therapy dose is defined by the ratio of fat calories to combined carbohydrate and protein calories. While a standard ketogenic diet typically uses a 3:1 to 4:1 ratio, kPN formulations rarely exceed a 2:1 ratio due to glycerol‐derived carbohydrates in intravenous lipid emulsions. If energy requirements are low, the ketogenic ratio may be even further reduced. In this case, a ratio of 1.5 was sufficient to induce ketosis and improve seizures, while supporting bowel recovery.

Initiating kPN requires a structured, stepwise approach (Table 2). As with any PN formulation, first determine daily energy and volume requirements. Energy needs can be estimated from prior caloric intake and growth trajectory. Resting energy expenditure can also be estimated using the Schofield equation and indirect calorimetry.^4^ Total fluid volume should be calculated using the Holliday–Segar method and further adjusted based on clinical status and fluid requirements. Dextrose should start at 5% and be weaned as close to 0% as the formulation permits. SMOF, a composite of soybean oil, medium‐chain triglycerides, olive oil, and fish oil, contains a small amount of glycerol (2.5 g per 100 mL of SMOF), which serves as an osmotic agent and stabilizer.^5^ Because glycerol can be metabolized to glucose, it contributes to the carbohydrate load and must be factored into carbohydrate calculations. Glucose should be maintained at 60–100 mg/dL with point‐of‐care monitoring every 8 h (more frequent if <70 mg/dL). Amino acids should meet full age‐specific protein requirements, adjusted for kidney function and catabolism.^6^ Start lipids at 0.05–1 g/kg/day and titrate to ketosis and energy needs. SMOF was used in this case because of its more rapid clearance than 100% soybean oil‐based lipid emulsions.^7^ The SMOF dosage was advanced up to 3.7 g/kg/day over 12 h (1.5 mL/kg/h). Although this exceeded the U.S. Food and Drug Administration‐recommended maximum infusion rate (0.75 mL/kg/h),^5^ serum triglyceride concentrations remained within an acceptable range (92–116 mg/dL), indicating adequate lipid clearance. Because the anticipated duration of PN was uncertain, SMOF was preferred over a pure soybean oil emulsion due to its lower phytosterol and omega‐6 fatty acid content in order to reduce the potential risk of PN‐associated liver disease. SMOF also contains a moderate amount of medium‐chain triglycerides, which have been proposed to exert antiseizure effects independent of ketosis by inhibiting glutamate receptors.^8^ Carnitine should be supplemented to promote lipid utilization. Electrolytes, multivitamins, and trace elements should follow guidelines.^9^ The final formulation should achieve therapeutic ketosis. Laboratory monitoring should include glucose, β‐OHB, comprehensive metabolic panel, lipase, and triglycerides. The use of kPN is generally safe, but high lipid content can cause nephrolithiasis, hypoglycemia, hyperlipidemia, and hypertransaminasemia.^3^ Despite prolonged use, our patient had no side effects, likely due to the slow and stepwise advancement of kPN.

**Table 2:** Nutritional advancement and monitoring during ketogenic parenteral nutrition.

Parameter	Recommendation
Energy/fluid	Perform baseline nutritional assessment for daily energy and fluid needs; advance dextrose, protein, and lipid to meet estimated or measured energy requirements.
Dextrose	Start at 5% solution; wean to lowest % available. Remove carbohydrates from medications if possible.
Protein	Start at full age‐specific protein requirement.
Lipid	Start at 0.5–1 g/kg; advance to highest possible amount based on ketosis and energy need. Supplement with carnitine to optimize lipid usage.
Glucose	Goal 60–120 mg/dL; monitor every 8 h and then daily once stable.
Labs	Daily, then weekly once stable:
	Beta‐hydroxybutyrate (goal >1.0 mmol/L)
	Comprehensive metabolic panel
	Lipase
	Triglyceride (goal <250 mg/dL in infants and <400 mg/dL in older children)

The need for prolonged bowel rest in this case stemmed from a duodenal perforation requiring parenteral ketogenic therapy. Duodenal perforation from pressure necrosis by the jejunal limb of a gastrojejunal tube is a recognized complication in small patients due to narrow bowel diameter and thickness.^10^ A systematic review of over 2700 procedures documented elevated risk in infants weighing <10 kg (perforation rate 3.1%, 95% confidence interval [CI] 1.1%–5.0%) versus 0.1% in children ≥10 kg (relative risk [RR] 9.4, 95% CI: 2.8–31.3).^10^ Our patient weighed 10 kg, where the risk of perforation is low but not eliminated. Newer low‐profile gastrojejunal tubes with a thinner and more flexible 8 Fr jejunal segment (Micro G‐JET®; Applied Medical Tech) are now used in smaller infants that may lower this risk.

## 4 CONCLUSION

In summary, kPN may be an effective and safe strategy for drug‐resistant epilepsy when enteral ketogenic diet administration is not feasible due to feeding intolerance or bowel rest. This case demonstrates the step‐wise initiation of safe long‐term kPN (>21 days) in a ketogenic diet‐naïve patient with refractory epilepsy who required prolonged post‐surgical bowel rest.

## CONFLICT OF INTEREST STATEMENT

The authors declare no conflicts of interest.

## ETHICS STATEMENT

Written permission was obtained from the patient's guardian to publish this case.

## Data Availability

Data sharing is not applicable to this article as no datasets were generated or analyzed during the current study.
